# Cone beam computed tomography evaluation of the relationship between atlantodental interval and skeletal facial morphology in adolescents^☆^

**DOI:** 10.1016/j.bjorl.2019.05.005

**Published:** 2019-06-18

**Authors:** Emre Cesur, Kaan Orhan, Melis Misirli, Burak Bilecenoglu

**Affiliations:** a Private Practice, Ankara, Turkey; b University of Leuven, Faculty of Medicine, Department of Imaging & Pathology, Leuven, Belgium; c Ankara University, Faculty of Dentistry, Department of DentoMaxillofacial Radiology, Ankara, Turkey; d Near East University, Faculty of Dentistry, Department of DentoMaxillofacial Radiology, Nicosia, Cyprus; e Ankara University, Faculty of Dentistry, Department of Anatomy, Ankara, Turkey

**Keywords:** Atlantodental interval, Cervical vertebrae, Facial morphology, CBCT, Intervalo atlantodental, Vértebra cervical, Morfologia facial, TCFC

## Abstract

**Introduction:**

In the pediatric population, computed tomography examination of the upper cervical spine plays an important role in the diagnosis of neurological injuries involving that region. Due to the interconnected nature of the craniofacial structures, a structural change in one is expected to also cause changes in the other structures.

**Objective:**

The aim of this study was to evaluate relationships between atlantodental interval, cervical vertebral morphology, and facial structure in healthy adolescents using cone beam computed tomography.

**Methods:**

Thirty subjects aged 14–20 years (10 males, mean age: 17.2 years; 20 females, mean age: 17.9 years) were included in the study. The anterior, lateral and posterior atlantodental intervals, and vertical and anteroposterior dimensions of the first and second cervical vertebrae were evaluated from cone beam computed tomography images. Facial morphology was evaluated using 7 parameters on lateral cephalometric cone beam computed tomography images and 6 parameters on posteroanterior images. The Mann–Whitney U test and Wilcoxon test were used for statistical analyses.

**Results:**

Comparisons between males and females showed that most parameters were larger in males, with significant differences in vertical facial dimensions (anterior lower face height: *p* = 0.05; anterior upper face height: *p* = 0.001), (distance between the most internal point of the frontozygomatic suture and midsagittal reference plane; *p* = 0.01), (the distance between the deepest point of the right alveolar maxillar process and midsagittal reference plane; *p* = 0.001), and C2 vertebral dimensions. The anterior and lateral atlantodental interval values correlated with maxilla position relative to the mandible angle, and the anterior atlantodental interval correlated with lower anterior facial height (*p* = 0.05). Dimensional measurements of the C1 and C2 vertebrae were correlated with both anterior facial heights and some posteroanterior parameters.

**Conclusion:**

Sagittal, vertical, and transverse facial dimensions and positions were strongly associated with C1 and C2 vertebral dimensions, and the maxillomandibular relationship may affect atlantodental interval. Therefore, including craniofacial features in assessment of the atlantodental area and vertebral distances in adolescents may be beneficial.

## Resumo

**Introdução:**

Na população pediátrica, a tomografia computadorizada da coluna cervical alta tem um importante papel no diagnóstico de lesões neurológicas que envolvem essa região. Devido à natureza interconectada das estruturas craniofaciais, espera-se que uma mudança estrutural em uma delas também cause alterações nas outras estruturas.

**Objetivo:**

O objetivo deste estudo foi avaliar as relações entre o intervalo atlantodental, a morfologia vertebral cervical e a estrutura facial em adolescentes saudáveis ​​com a tomografia computadorizada de feixe cônico.

**Método:**

Trinta indivíduos entre 14 e 20 anos (10 homens, média de idade: 17,2 anos; 20 mulheres, média de idade: 17,9 anos) foram incluídos no estudo. O intervalo atlantodental anterior, lateral e posterior e as dimensões vertical e anteroposterior da primeira e segunda vértebras cervicais foram avaliados a partir de imagens de tomografia computadorizada de feixe cônico. A morfologia facial foi avaliada utilizando-se 7 parâmetros em imagens de tomografia computadorizada de feixe cônico cefalométricas laterais e 6 parâmetros em imagens posteroanteriores. O teste U de Mann-Whitney e o teste de Wilcoxon foram utilizados para as análises estatísticas.

**Resultados:**

As comparações entre homens e mulheres mostraram que em sua maioria os parâmetros foram maiores no sexo masculino, com diferenças significantes nas dimensões faciais verticais (altura facial anterior inferior:*p* = 0,05; altura facial anterior superior: *p* = 0,001), distância entre o ponto mais interno da sutura fronto-zigomática e plano de referência médio-sagital; *p* = 0,01distância entre o ponto mais profundo do processo alveolar do maxilar direito e o plano de referência médio-sagital; *p* = 0,001) e as dimensões do corpo vertebral C2. O intervalo atlantodental anterior e lateral correlacionaram-se com o ângulo da posição da maxila em relação à mandíbula e o intervalo atlantodental anterior correlacionou-se com altura facial anterior inferior (*p* = 0,05). Medidas das dimensões das vértebras C1 e C2 foram correlacionadas com as alturas faciais anteriores e alguns parâmetros póstero-anteriores.

**Conclusão:**

As dimensões e posições faciais sagitais, verticais e transversais foram fortemente associadas às dimensões dos corpos vertebrais C1 e C2 e a relação maxilomandibular pode afetar o intervalo atlantodental. Portanto, incluir características craniofaciais na avaliação da área atlantodental e das distâncias vertebrais em adolescentes pode ser benéfico.

## Introduction

The craniocervical junction has a complex structural and functional anatomy and is vulnerable to trauma and injuries due to its high mobility and flexibility.^1^, ^2^ The AtlantoDental Interval (ADI) is defined as the distance between the anterior edge of the odontoid process of the second cervical vertebra and the posteroinferior edge of the anterior arch of the atlas, and is used to identify atlantoaxial segment instability.^3^

Measurements of the Lateral (LADI) and Anterior (AADI) portions of the ADI are frequently used to detect trauma in the associated regions. However, the sensitivity of LADI is still under debate, because LADI asymmetry is also seen in healthy individuals and those asymptomatic following trauma.^4^, ^5^ Assessment of ADI may be affected by factors such as age and degenerative changes, gender, and habitual posture.^3^ Studies in the pediatric population have revealed differences in cervical spine morphology compared to adults and variation in the criteria that should be considered when evaluating trauma and injuries in this region.^2^, ^6^, ^7^

Due to the interconnected nature of the craniofacial structures, a structural change in one is expected to also cause changes in the other structures.^8^ Orthodontic studies have examined relationships between not only the jaws and teeth, but have also included the cervical vertebrae for various reasons.^9^ Cervical vertebral analysis is an important tool in orthodontic diagnosis and treatment, especially when evaluating skeletal maturation.^10^, ^11^ Previous studies have also shown that patients with different malocclusions and individuals with cleft lip/palate may present various vertebral anomalies.

Cephalometric radiographs have become one of the most valuable tools in orthodontic treatment planning and the evaluation of treatment outcomes and patient growth.^12^ However, the complex anatomy of the atlantoaxial region precludes adequate evaluation by conventional radiography, which involves anatomic superposition and reduced measurement accuracy. For this reason, cone beam computed tomography (CBCT) is increasingly preferred in current practice.^4^, ^13^

Although previous studies have demonstrated a relationship between cervical vertebral anomalies and skeletal facial morphology, there are no studies in the literature on the relationship between atlantodental morphology and skeletal facial structures. In light of this information, this study was conducted to evaluate the relationship between atlantodental interval and morphology and the facial structures of healthy adolescents using CBCT.

## Methods

### Patient selection

This retrospective study was done using CBCT images of patients who presented to the Faculty of Dentistry of Ankara University and Near East University for various reasons. Ethical approval for the study was obtained from the Near East University Faculty of Dentistry Ethics Committee (YDU/2018/54-497).

Thirty patients aged 14–20 years (10 males, mean age: 17.2 years; 20 females, mean age: 17.9 years) were included in the study. The patients’ CBCT images were selected from among those obtained for various dental procedures (impacted tooth extraction, implant placement, etc.).

Inclusion criteria were as follows:

1) Being at or younger than 20 years old;
2) Not having orthodontic treatment or functional orthopaedic treatment or orthognathic surgery during/before the CBCT procedure;
3) Not having a craniofacial congenital anomaly such as cleft lip and plate, syndromes affecting craniofacial region, etc.

Exclusion criteria for the study were:

1) Trauma or trauma history affecting craniofacial region and causing malformations and the lack of growth of jaws,
2) History of cervical surgery or any procedure involving the cervical region,
3) Any condition related to a congenital anomaly or syndrome (cleft lip and palate or any other condition/syndrome associated with craniofacial region).

### CBCT measurements

CBCT examinations were performed by using a NewTom 3G (Quantitative Radiology srl, Verona, Italy). All images were recorded at 120 kVP and 3–5 mA using a 12 inch field of view, axial slice thickness of 0.3 mm, and isotropic voxels. CBCT images were exported in 512 × 512 matrix Digital Imaging and Communications in Medicine (DICOM) format. Maxilim version 2.3.0 (Medicim, Mechelen, Belgium) software program was used to generate 3D surface models and take orthodontic measurements. Vertical and horizontal measurements of the vertebrae were done from the CBCT data set linked to the 3D rendering software (Anatomage, Invivo 5.2).

**Figure 1:**
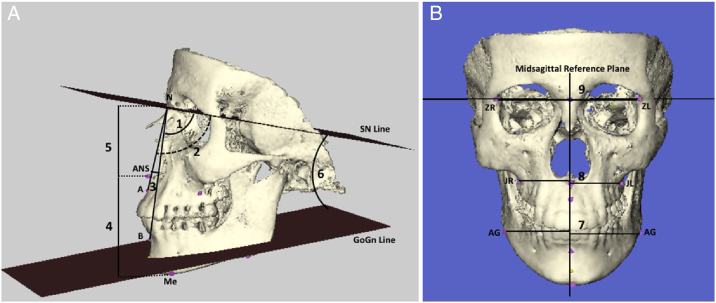
A) Lateral cephalometric measurements: (1) SNA (°): the postero-inferior angle between anterior cranial base and nasion-point A line; (2) SNB (°): the postero-inferior angle between anterior cranial base and nasion-point B line; (3) ANB (°): the angle between nasion-point A and nasion-point B lines; (4) ANS-Me (mm): anterior lower facial height; (5) N-ANS (mm): anterior upper facial height; (6) GoGn/SN (°), the angle between anterior cranial base and the mandibular plane; (B) Posteroanterior measurements: (7) GA-MSR (mm): linear distance between antegonial notch and midsagittal reference plane; (8) JL-MSR (mm): linear distance between the deepest point of the right alveolar maxillar process and midsagittal reference plane; (9) ZL-MSR (mm): linear distance between the most internal point of the frontozygomatic suture and midsagittal reference plane.

Landmarks used for the evaluation of facial structures are shown in Table 1. From lateral cephalometric images, we evaluated maxilla position relative to the cranial base (SNA angle), mandible position relative to the cranial base (SNB angle), maxilla position relative to the mandible (ANB angle), lower anterior facial height (ANS-Me distance), upper anterior facial height (N-ANS distance), and the angle between the mandibular plane and the cranial base (GoGn/SN angle). Considering possible asymmetry in the gonion region of the mandible, left and right GoGn/SN angles were determined (Fig. 1).

**Table 1:** Cephalometric sagittal and posteroanterior landmarks.

Sagittal landmarks	
Nasion (N)	The most anterior point of the front nasal suture
Sella (S)	The midpoint of sella turcica
A	The deepest point of concavity on the maxilla between ANS and prosthion
B	The deepest point of concavity on the mandibular symphysis between infradentale and pogonion
ANS	The most anterior point of anterior nasal spine
Me	The midpoint on the inferior border of the mental protuberances
Gonion (Go)	Point of intersection of the ramus plane and the mandibular plane
Gnnathion (Gn)	The most anteroinferior point on the symphysis
Posteroanterior landmarks	
Crista Galli (CG)	The most upper point of crista galli
ZR/ZL	The most internal point of the frontozygomatic suture (right and left)
JR/JL	Intersection of the maxillar tuberosity with the zygomatic process contour (right and left)
AG	Highest point in the antegonial notch

In order to perform posteroanterior facial analyses, a midsagittal reference plane (MSR) was determined for frontal images by drawing a vertical line from the crista galli peak to the plane connecting the ZL (the most internal point of the left frontozygomatic suture) and ZR points (the most internal point of the right frontozygomatic suture). Measurements of AG-MSR (the distance between the right antegonial notch and midsagittal reference plane) and GA-MSR (the distance between the left antegonial notch and midsagittal reference plane) were made to evaluate mandibular asymmetry, JR-MSR (the distance between the deepest point of the right alveolar maxillar process and midsagittal reference plane) and JL-MSR (the distance between the deepest point of the left alveolar maxillar process and midsagittal reference plane) to evaluate maxillary symmetry, and ZR-MSR and ZL-MSR to evaluate upper facial symmetry (Fig. 1).

**Figure 2:**
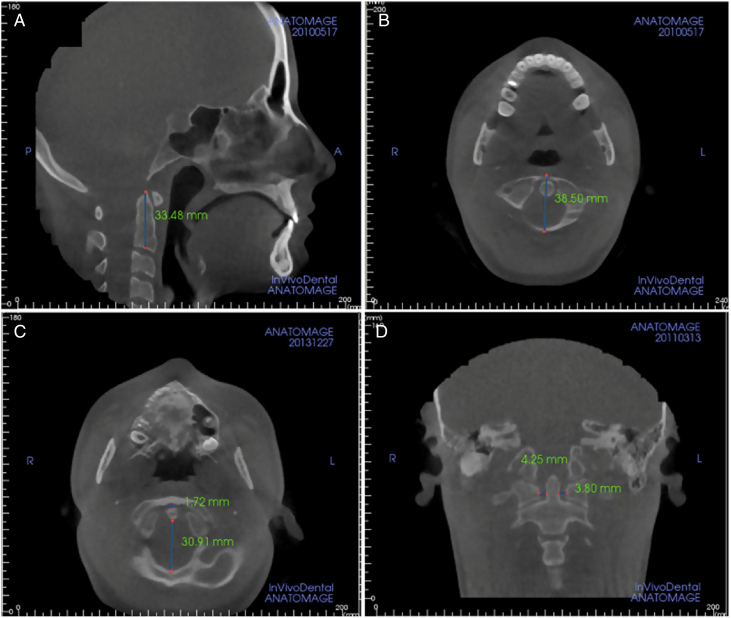
(A) Vertical dimension measurement of cervical vertebra; (B) Anteroposterior dimension measurement of cervical vertebra; (C) measurements of anterior and posterior atlantodental interval; (D) measurements of lateral atlantodental interval.

Eight CBCT parameters were evaluated in our analysis of the atlantodental region. Atlas (C1) and axis (C2) morphology were evaluated using C1 vertical length (C1 Ver) and anteroposterior length (C1 AP), C2 vertical length (C2 Ver) and anteroposterior length (C2 AP). The ADI was evaluated by measuring AADI (the distance between the anterior edge of dens and the posterior edge of the anterior arch of the atlas), PADI (posterior atlantodental intervalthe, distance between the posterior edge of the dens and the anterior edge of the posterior arch of the atlas), and LADI (Fig. 2).

### Statistical analysis

CBCTs of a total of 15 individuals were randomly selected and examined again approximately 4 weeks after the initial measurements to determine repeatability of the measurements. The study data were statistically analyzed using SPSS v.17.0 software package (SPSS Inc., Chicago, IL, USA). Based on our sample size, Shapiro–Wilk test was used to determine whether the data followed a normally distribution (*p* < 0.05 indicated non-normal distribution, *p* > 0.05 indicated normal distribution). The Mann–Whitney U test was used to evaluate differences in non-normally distributed variables between the groups. The Wilcoxon test was used to evaluate differences in dependent variables with non-normal distribution. Spearman’s correlation coefficient was used to examine relationships between non-normally distributed variables.

## Results

Males showed significantly larger values for ANS/Me (*p* = 0.05), N/ANS (*p* = 0.001), JR-MSR (*p* = 0.001), and ZL-MSR (*p* = 0.01). In atlantodental measurements, males also had significantly larger C2 Ver (*p* = 0.05) and C2 AP (*p* = 0.001) values (Table 2).

**Table 2:** Comparison of measurements between genders by Mann–Whitney U test.

		Gender		Mann–Whitney U test	
		n	Mean ± SD	z	*p*
Cephalometric lateral measurements					
SNA (°)	M	10	85.62 ± 6.40	−1.496	0.135
	F	20	82.35 ± 3.18		
SNB (°)	M	10	81.50 ± 5.50	−1.364	0.173
	F	20	78.73 ± 3.60		
ANB (°)	M	10	4.79 ± 2.25	−0.440	0.660
	F	20	4.15 ± 1.93		
ANS-Me (mm)	M	10	65.48 ± 5.61	−2.332	0.020^a^
	F	20	59.60 ± 6.10		
N-ANS (mm)	M	10	53.39 ± 4.23	−3.278	0.001^c^
	F	20	47.65 ± 3.06		
GoGn/SN° (right)	M	10	24.09 ± 5.17	−1.232	0.218
	F	20	26.13 ± 5.65		
GoGn/SN° (left)	M	10	23.31 ± 4.81	−1.320	0.187
	F	20	25.73 ± 8.37		
Postero-anterior measurements (mm)					
AG-MSR (right)	M	10	43.04 ± 5.77	−1.122	0.262
	F	20	40.76 ± 2.84		
AG-MSR (left)	M	10	43.67 ± 5.08	−1.562	0.118
	F	20	41.60 ± 3.12		
JR-MSR (right)	M	10	31.89 ± 1.30	−3.215	0.001^c^
	F	20	29.32 ± 1.99		
JL-MSR (left)	M	10	30.32 ± 2.74	−1.828	0.068
	F	20	28.44 ± 2.05		
ZR-MSR (right)	M	10	47.22 ± 2.84	−2.113	0.035
	F	20	45.00 ± 2.10		
ZL-MSR (left)	M	10	47.95 ± 2.65	−2.575	0.010^b^
	F	20	45.31 ± 2.31		
Atlantodental interval measurements					
C1 Ver	M	10	10.01 ± 1.92	−1.211	0.226
	F	20	9.10 ± 1.29		
C1 AP	M	10	44.76 ± 3.35	−2.157	0.031
	F	20	42.20 ± 4.03		
C2 Ver	M	10	34.55 ± 4.01	−2.488	0.013^a^
	F	20	32.23 ± 2.71		
C2 AP	M	10	46.75 ± 3.12	−3.324	0.001^c^
	F	20	41.89 ± 3.45		
Anterior ADI	M	10	1.66 ± 0.63	−1.763	0.078
	F	20	1.28 ± 0.46		
Posterior ADI	M	10	21.04 ± 2.54	−1.276	0.202
	F	20	20.04 ± 3.16		
Lateral ADI (right)	M	10	4.09 ± 0.85	−0.727	0.467
	F	20	3.71 ± 0.90		
Lateral ADI (left)	M	10	3.71 ± 0.40	−1.082	0.279
	F	20	3.55 ± 0.98		
	F	20	32.23 ± 2.71		

M, male; F, female; SD, standard deviation.

a *p* *=* 0.05.

b *p* = 0.01.

c *p* = 0.001.

In cephalometric and posteroanterior images, comparison of right and left side measurements showed that JR-MSR values were significantly greater than JL-MSP values (*p* = 0.01) (Table 3).

**Table 3:** Comparison of right/left side cephalometric lateral and posteroanterior measurements by Wilcoxon test.

					Wilcoxon test	
	n	Mean	Min.	Max. ± SD	z	*p*
Cephalometric lateral measurements (°)						
GoGn/SN (right)	30	25.5	11.6	35.4 ± 5.5	−0.113	0.910
Gogn/SN (left)	30	24.92	0.00	36.10 ± 7.38		
Postero-anterior measurements (mm)						
AG-MSR (right)	30	41.5	31.9	52.1 ± 4.1	−1.152	0.249
AG-MSR (left)	30	42.3	34.5	50.4 ± 3.9		
JR-MSR (right)	30	30.2	25.2	33.6 ± 2.2	−2.664	0.008^a^
JL-MSR (left)	30	29.1	24.9	35.4 ± 2.4		
ZR-MSR (right)	30	45.7	40.8	51.9 ± 2.6	−1.728	0.084
ZL-MSR (left)	30	46.2	41.2	50.8 ± 2.7		

Min, minimum; Max, maximum; SD, standard deviation.

a *p* = 0.01.

Evaluation of correlations between atlantodental parameters in cephalometric lateral and posteroanterior images revealed the following (Table 4):

**Table 4:** Correlations between cephalometric/anteroposterior facial measurements and atlantodental measurements.

		C1 Ver	C1 AP	C2 Ver	C2 AP	Anterior ADI	Posterior ADI	Lateral ADI (right)	Lateral ADI (left)
Cephalometric lateral measurements									
SNA (°)	r	−0.240	−0.156	−0.013	0.150	0.038	−0.244	−0.212	0.084
	*p*	0.202	0.410	0.944	0.428	0.841	0.194	0.260	0.659
	N	30	30	30	30	30	30	30	30
SNB (°)	r	−0.034	−0.071	0.190	0.067	−0.101	−0.082	−0.296	−0.143
	*p*	0.858	0.708	0.316	0.727	0.595	0.667	0.113	0.450
	N	30	30	30	30	30	30	30	30
ANB (°)	r	−0.266	0.056	−0.293	0.148	0.429^a^	0.028	0.180	0.420^a^
	*p*	0.156	0.768	0.116	0.436	0.018	0.881	0.341	0.021
	N	30	30	30	30	30	30	30	30
ANS-Me (mm)	r	0.427^a^	0.346	0.448^a^	0.457^a^	0.436^a^	0.022	0.140	0.009
	*p*	0.019	0.061	0.013	0.011	0.016	0.909	0.460	0.960
	N	30	30	30	30	30	30	30	30
N-ANS (mm)	r	0.468^b^	0.452^a^	0.557^c^	0.618^c^	0.290	0.230	0.274	−0.107
	*p*	0.009	0.012	0.001	0.000	0.120	0.221	0.143	0.572
	N	30	30	30	30	30	30	30	30
GoGn/SN° (right)	r	0.115	0.207	−0.119	−0.110	0.353	0.085	−0.037	0.200
	*p*	0.546	0.271	0.530	0.563	0.056	0.656	0.846	0.290
	N	30	30	30	30	30	30	30	30
GoGn/SN° (left)	r	0.020	0.297	−0.083	−0.172	0.128	0.181	0.007	0.122
	*p*	0.917	0.111	0.663	0.364	0.500	0.339	0.970	0.522
	N	30	30	30	30	30	30	30	30
Postero-anterior measurements									
AG-MSR (right)	r	0.377^a^	0.134	0.459^a^	0.643^c^	0.010	0.197	0.212	−0.133
	*p*	0.040	0.479	0.011	0.000	0.958	0.296	0.262	0.485
	N	30	30	30	30	30	30	30	30
AG-MSR (left)	r	0.452^a^	0.195	0.647^c^	0.424^a^	0.227	0.214	0.186	−0.115
	*p*	0.012	0.302	0.000	0.019	0.228	0.257	0.326	0.544
	N	30	30	30	30	30	30	30	30
JR-MSR (right)	r	0.161	0.090	0.588^c^	0.693^c^	0.207	0.002	0.318	−0.256
	*p*	0.395	0.636	0.001	0.000	0.272	0.992	0.087	0.171
	N	30	30	30	30	30	30	30	30
JL-MSR (left)	r	0.230	0.023	0.648^c^	0.372^a^	0.226	−0.213	0.109	−0.177
	*p*	0.221	0.906	0.000	0.043	0.230	0.259	0.565	0.351
	N	30	30	**30**	**30**	30	30	30	30
ZR-MSR (right)	r	0.378^a^	0.397^a^	0.342	0.520^b^	0.298	0.345	0.242	0.071
	*p*	0.040	0.030	0.064	0.003	0.109	0.061	0.198	0.709
	N	30	30	30	30	30	30	30	30
ZL-MSR (left)	r	0.390^a^	0.186	0.442^a^	0.526^b^	0.195	0.145	0.261	−0.037
	*p*	0.033	0.326	0.015	0.003	0.301	0.445	0.164	0.847
	N	30	30	30	30	30	30	30	30

a *p* = 0.05.

b *p* = 0.01.

c *p* = 0.00.

A moderate positive correlation was observed between ANB angle and AADI and LADI (left) values (*p* = 0.05). Lower anterior height (ANS/Me) showed a moderate positive correlation with C1 Ver, C2 Ver, C2 AP, and AADI (*p* = 0.05). Upper anterior face height (N-ANS) was also positively correlated with C1 Ver (*p* = 0.01), C1 AP (*p* = 0.05), C2 Ver (*p* = 0.01), and C2 AP (*p* = 0.01) values.

Changes in AG-MSR and GA-MSP values were positively correlated with changes in C1 Ver, C2 Ver, and C2 AP values (*p* = 0.05). JR-MSR and JL-MSP values also showed significant positive correlation with C2 Ver (*p* = 0.01) and C2 AP (right: *p* = 0.01; left: *p* = 0.05). ZR-MSR was positively correlated with C1 Ver, C1 AP (*p* = 0.05) and C2 AP (*p* = 0.01), while ZL-MSR was correlated with C1 Ver, C2 Ver (*p* = 0.05), and C2 AP (*p* = 0.01).

## Discussion

The atlantodental interval measurement is the most common method used to evaluate the stability of the atlantoaxial joint and transverse ligament. In the pediatric population, CT examination of the upper cervical spine plays an important role in the diagnosis of neurological injuries involving that region.^2^ Previous studies have shown that ADI measurements may show age-related changes.^2^, ^3^, ^5^, ^6^^,^^14^ Osmotherly et al. reported that ADI value may decrease with older age, especially in the presence of minor clinical instability, and emphasized that age must be considered.^3^ Previous studies examining craniofacial anomalies and vertebral morphology suggest a possible relationship between these structures.^11^, ^15^, ^16^, ^17^, ^18^ However, these studies were based on 2D images, and ADI measurements were not evaluated. Therefore, our study is the first to evaluate the relationship between facial morphology, vertebral dimensions, and ADI using 3D images.

In this study, comparisons of cephalometric, posteroanterior, and vertebral measurements from the CBCT images of males and female adolescents showed that most parameters were larger in males, with significant sex differences in vertical dimensions (ANS-Me, N-ANS), ZR-MSR and JR-MSR values, and C2 vertebral dimensions. Baccetti et al. studied the effect of sexual dimorphism on craniofacial morphology among class III individuals and reported that males had relatively larger maxillary, mandibular, and anterior facial height compared to females after the onset of puberty, especially boys over the age of 13.^19^ Similarly, Ursi et al. reported that after the age of 14, maxillary and mandibular dimensions remained stable in females but increased significantly in males.^20^ All of the individuals included in our study were between 14 and 20 years of age. Therefore, considering the age group, dimensional differences between the sexes observed in this study should be considered a physiological condition.

Numerous studies by biologists, anatomists, and anthropologists have shown that asymmetry of form, function, and proportions is common among mammals and/or invertebrates in nature.^21^ Thiesen et al. reported that due to the effects of both environmental factors and biological factors on symmetry, facial symmetry is very rare.^22^ However, Shah and Joshi found that even in patients with excellent occlusion and acceptable facial esthetics, the right side of the face was significantly larger than the left side.^21^ Type of malocclusion and facial asymmetry were not considered when selecting patients for inclusion in our study. However, consistent with previous studies, our separate evaluation of right and left side measurements showed that JR-MSR values were greater than JL-MSR; in other words, we observed asymmetry in the maxillary region (Table 3).

The ANB angle, which represents the maxillomandibular relationship, was positively correlated with AADI and left LADI values in our study. Aranatasi et al. observed differences between skeletal Class I, II and III individuals in terms of cervical vertebral anomalies.^11^ Arntsen and Sonnesen reported that deviation in cervical vertebral column morphology may be common (28%) among patients with skeletal maxillary overjet.^16^

In addition, the relationship between anterior facial height and vertical and anteroposterior vertebral dimensions was noteworthy. Facial measurements on posteroanterior images also showed an association between posteroanterior measurements and cervical vertebral dimensions, which was more prominent in C2. Gupta et al. claimed that there was a relationship between C2 Ver and maxillary and mandibular length, especially in patients with vertical growth patterns.^23^

Our study demonstrates that the sagittal, vertical, and transverse dimensions and positions of the face are strongly associated with first and second cervical vertebral dimensions, and indicates that the maxillomandibular relationship may affect ADI. According to Huggare, horizontal and vertical growth of the first cervical vertebra is regulated by different mechanisms.^24^ An increase in diameter occurs via intervertebral synchondrosis, while increase in height occurs by appositional growth influenced by muscle function. Therefore, growth of the cervical vertebrae occurs not only as a result of natural growth, but also in association with function, head posture, and craniofacial structural features.^24^, ^25^ Furthermore, the relationship between deviations in cervical vertebral column morphology and craniofacial morphology may be associated with early embryogenesis. During this period, the notochord is involved in the development of both the cervical vertebra and the basilar part of the occipital bone. Therefore, a deviation in notochord development may affect the development of both of these structures. Due to the connection between the jaws and the cranial base and the interactions of the facial skeletal system, cervical vertebral morphology may be associated with craniofacial features.^15^, ^25^, ^26^

## Conclusion

Based on the results of our study, AADI may be associated with ANB angle and lower anterior facial height (ANS-Me) in adolescents. Thus, it should be kept in mind during post-traumatic evaluation that increases in maxillomandibular sagittal distance and lower anterior facial height may affect the ADI measurements. Our findings also demonstrated a mutual interaction between anterior facial heights and PA facial distances and vertebral dimensions.

Therefore, considering not only age and gender but also craniofacial features in the assessment of the atlantodental region and vertebral distances in adolescents may benefit radiologists and clinicians during the diagnostic process. Further studies with larger samples and more detailed measurements are needed to reach more definitive conclusions on this topic.

## Funding

This research did not receive any specific grant from funding agencies in the public, commercial, or not-for-profit sectors.

## Conflicts of interest

The authors declare no conflicts of interest.
